# Supplementary material for: Low levels of Plasmodium falciparum parasitaemia among patients receiving antiretroviral therapy at the treatment centre of the Regional Hospital Bamenda, Northwest Cameroon
Source: PLoS One. 2025 Jan 29;20(1):e0317120. doi: 10.1371/journal.pone.0317120 (PMC11778629; doi:10.1371/journal.pone.0317120)
Supplement: Attachment — Submitted filename: PONE-D-24-04976 - Reviewed Copy.pdf [file pone.0317120.s002.pdf]

PLOS ONE

Low levels of Plasmodium falciparum parasitemia among patients receiving antiretroviral therapy at the treatment center of the Regional Hospital Bamenda, Northwest Cameroon

--Manuscript Draft--

**Manuscript Number:**	PONE-D-24-04976
**Article Type:**	Research Article
**Full Title:**	Low levels of Plasmodium falciparum parasitemia among patients receiving antiretroviral therapy at the treatment center of the Regional Hospital Bamenda, Northwest Cameroon
**Short Title:**	Low levels of malaria parasitemia in HIV/AIDS patients in RHB, Cameroon
**Corresponding Author:**	BISONG CALVIN EBAI, Ph.D.<br>UBa: University of Bamenda<br>Bambili, North West Region CAMEROON
**Keywords:**	Plasmodium falciparum parasitemia; HIV/AIDS; antiretroviral therapy; treatment; treatment center; Regional hospital Bamenda.
**Abstract:**	This research is carried out in line with my area of research in malaria and other parasitic diseases. This particular study has diagnosed the prevalence and density of malaria among HIV/AIDS patients receiving antiretroviral treatment in the treatment center of the Regional Hospital Bamenda in the Northwest of Cameroon. Malaria and HIV are two infections that continue to hit hard on the health and economy of countries in sub-Saharan Africa. So findings form our study could contribute to shaping policy on the integrated management of HIV/AIDS and malaria in Cameroon and other countries. This research meets the scope of the PLOSONE Global Public Health Journal so we recommend it should be published there.
**Order of Authors:**	BISONG CALVIN EBAI, Ph.D.
	Cedric Yamssi, Ph.D.
	f Nguemaïm Ngoufo, Ph.D.
	Nicaise Tchoffo Ngouemeta
	Nfor Omarine Nlinwe, Ph.D.
	Helen Kuokuo Kimbi, Ph.D.
**Additional Information:**	
**Question**	**Response**
**Financial Disclosure**<br><br>Enter a financial disclosure statement that describes the sources of funding for the work included in this submission. Review the submission guidelines for detailed requirements. View published research articles from *PLOS ONE* for specific examples.<br><br>This statement is required for submission and **will appear in the published article** if the submission is accepted. Please make sure it is accurate.	The author(s) received no specific funding for this work.

Powered by Editorial Manager® and ProduXion Manager® from Aries Systems Corporation

**Funded studies**<br>Enter a statement with the following details:<br>• Initials of the authors who received each award<br>• Grant numbers awarded to each author<br>• The full name of each funder<br>• URL of each funder website<br>• Did the sponsors or funders play any role in the study design, data collection and analysis, decision to publish, or preparation of the manuscript?<br><br>Did you receive funding for this work?	
**Competing Interests**<br><br>Use the instructions below to enter a competing interest statement for this submission. On behalf of all authors, disclose any competing interests that could be perceived to bias this work—acknowledging all financial support and any other relevant financial or non-financial competing interests.<br><br>This statement is required for submission and **will appear in the published article** if the submission is accepted. Please make sure it is accurate and that any funding sources listed in your Funding Information later in the submission form are also declared in your Financial Disclosure statement.<br><br>View published research articles from *PLOS ONE* for specific examples.	The authors have declared that no competing interests exist.

Powered by Editorial Manager® and ProduXion Manager® from Aries Systems Corporation

**NO authors have competing interests**<br><br>Enter: *The authors have declared that no competing interests exist.*<br><br>**Authors with competing interests**<br><br>Enter competing interest details beginning with this statement:<br><br>*I have read the journal's policy and the authors of this manuscript have the following competing interests: [insert competing interests here]*<br><br>* typeset	
**Ethics Statement**<br><br>Enter an ethics statement for this submission. This statement is required if the study involved:<br><br>• Human participants<br>• Human specimens or tissue<br>• Vertebrate animals or cephalopods<br>• Vertebrate embryos or tissues<br>• Field research<br><br>Write "N/A" if the submission does not require an ethics statement.<br><br>General guidance is provided below. Consult the submission guidelines for detailed instructions. **Make sure that all information entered here is included in the Methods section of the manuscript.**	Ethical clearance was obtained from the Institutional Review Board of the University of Bamenda hosted by the Faculty of Health Sciences.<br>Written informed consent was obtained from participants and data was analyzed anonymously.<br>Statement from Institutional Review Board: "The Ethical review committee of the University of Bamenda, after a study of your project proposal, and in keeping with existing regulations, hereby has no objection on ethical grounds provided the project is carried out as described in the protocol submitted to the committee/Board. The attestation is non-transferable. Any changes to the protocol and/or personnel must get the approval of the Ethical committee/Institutional Review Board otherwise the clearance will be nullified".

Powered by Editorial Manager® and ProduXion Manager® from Aries Systems Corporation

**Format for specific study types**<br><br>**Human Subject Research (involving human participants and/or tissue)**<br>• Give the name of the institutional review board or ethics committee that approved the study<br>• Include the approval number and/or a statement indicating approval of this research<br>• Indicate the form of consent obtained (written/oral) or the reason that consent was not obtained (e.g. the data were analyzed anonymously)<br><br>**Animal Research (involving vertebrate animals, embryos or tissues)**<br>• Provide the name of the Institutional Animal Care and Use Committee (IACUC) or other relevant ethics board that reviewed the study protocol, and indicate whether they approved this research or granted a formal waiver of ethical approval<br>• Include an approval number if one was obtained<br>• If the study involved *non-human primates*, add *additional details* about animal welfare and steps taken to ameliorate suffering<br>• If anesthesia, euthanasia, or any kind of animal sacrifice is part of the study, include briefly which substances and/or methods were applied<br><br>**Field Research**<br><br>Include the following details if this study involves the collection of plant, animal, or other materials from a natural setting:<br>• Field permit number<br>• Name of the institution or relevant body that granted permission	
**Data Availability**<br><br>Authors are required to make all data underlying the findings described fully available, without restriction, and from the time of publication. PLOS allows rare exceptions to address legal and ethical concerns. See the PLOS Data Policy and FAQ for detailed information.	Yes - all data are fully available without restriction

Powered by Editorial Manager® and ProduXion Manager® from Aries Systems Corporation

A Data Availability Statement describing where the data can be found is required at submission. Your answers to this question constitute the Data Availability Statement and **will be published in the article**, if accepted.<br><br>**Important:** Stating 'data available on request from the author' is not sufficient. If your data are only available upon request, select 'No' for the first question and explain your exceptional situation in the text box.<br><br>Do the authors confirm that all data underlying the findings described in their manuscript are fully available without restriction?	
**Describe where the data may be found in full sentences. If you are copying our sample text, replace any instances of XXX with the appropriate details.**<br><br>• If the data are **held or will be held in a public repository**, include URLs, accession numbers or DOIs. If this information will only be available after acceptance, indicate this by ticking the box below. For example: *All XXX files are available from the XXX database (accession number(s) XXX, XXX.)*.<br>• If the data are all contained **within the manuscript and/or Supporting Information files**, enter the following: *All relevant data are within the manuscript and its Supporting Information files.*<br>• If neither of these applies but you are able to provide **details of access elsewhere**, with or without limitations, please do so. For example:<br><br>*Data cannot be shared publicly because of [XXX]. Data are available from the XXX Institutional Data Access / Ethics Committee (contact via XXX) for researchers who meet the criteria for access to confidential data.*<br><br>*The data underlying the results presented in the study are available from (include the name of the third party*	The dataset is available from the dryad database (accession number(s) https://doi.org/10.5061/dryad.d51c5b0b3

Powered by Editorial Manager® and ProduXion Manager® from Aries Systems Corporation

and contact information or URL).<br>• This text is appropriate if the data are owned by a third party and authors do not have permission to share the data.<br><br>* typeset	
Additional data availability information:	

Manuscript

Click here to access/download;Manuscript;Low levels of MP in HIV_AIDS patients in TC_RHBCAM.docx

Low levels of *Plasmodium falciparum* parasitemia among patients receiving antiretroviral therapy at the treatment center of the Regional Hospital Bamenda, Northwest Cameroon

Authors and affiliations:

Calvin Bisong Ebai[1*], Cedric Yamssi[2], Flore Nguemaïm Ngoufo[1,2], , Nicaise Ngouemeta Tchoffo[1], Nfor Omarine Nlinwe[1], and Helen Kuokuo Kimbi[2,3]

1: Department of Medical Laboratory Science, Faculty of Health Sciences, University of Bamenda, P.O. Box 39, Bambili, North West Region, Cameroon.

2: Department of Biomedical Sciences, Faculty of Health Sciences, University of Bamenda, P.O. Box 39, Bambili, North West Region, Cameroon.

3: Department of Animal Biology and Conservation, Faculty of Science, University of Buea, P.O. Box 63, Buea, South West Region, Cameroon.

*: Corresponding author (ebaipi2@yahoo.com)

Abstract

Background: Malaria and HIV are leading causes of death in Africa including Cameroon. Antiretroviral therapy is expected to boost immunity and reduce vulnerability to opportunistic infections. ~~That notwithstanding,~~ reports on comorbidities including malaria are common in Cameroon.

Objectives: ~~The objectives of this study were to~~ determine the prevalence and density of malaria parasitaemia, clinical manifestations and factors associated with malaria parasitaemia among HIV patients on ~~antiretroviral therapy.~~

Methods: It was a ~~hospital-based~~ cross-sectional study among HIV patients on ART at the Regional Hospital Bamenda. A pre-tested questionnaire was used to collect data. Capillary blood

was used for malaria parasite diagnosis and to determine white blood cell count. Data was analysed using SPSS version 20.

Results: The study included 181 participants. The overall prevalence of malaria ~~parasite~~ was 9.4%. Although there were no significant statistical differences, the following trends were observed in the results: 55-71 age group (14%) was most infected with malaria parasite; the highest prevalence ~~was~~ among those on the 8A first-line ART (10.3%, 14/122); ($\chi 2$=1.03, p=0.96), only participants in the 8A and 12A ART protocols were infected; parasite prevalence was higher in patients with detectable viral load (14.3%, 3/21), (OR=.0.57 (CI: 0.15-2.2), p=0.41), GMPD~~S~~ varied from 45.7-53.3-±23.1/µl of blood.

Conclusion: Low ~~levels of~~ malaria parasite prevalence and density were detected amongst HIV patients receiving ART. The prevalence of malaria parasitaemia and parasite density was similar despite variations among the patients. A systematic malaria test could be helpful to avert morbidity and improve the general health of patients on ART.

Keywords: *Plasmodium falciparum parasitaemia, HIV, antiretroviral therapy, treatment centre and Regional hospital Bamenda.*

1. Introduction

Malaria and HIV are two leading causes of death in Africa including Cameroon [1]. Malaria is a febrile disease caused by parasites of the genus *Plasmodium*. Five species infect humans present~~ing~~ with varied manifestations based on ~~their~~ interaction with the immune system ~~of the infected individual. These species include *Plasmodium falciparum, P. malariae, P. ovale, P. vivax* and *P. knowlesi*.~~ *P. knowlesi* ~~is~~ the most recent species identified in south eastern Asia as a parasite of macaques ~~which causes malaria in humans~~ similar to that caused by *P. falciparum* [2]~~. Malaria caused by *P. falciparum* is reported to be~~ the most severe ~~and~~ responsible for majority of deaths related to malaria [3]. It is the most common species in sub-Saharan Africa (SSA) [4] where the greatest burden of malaria is reported [5]. Malaria parasites are transmitted through the bites of an infected female *Anopheles* mosquito during its blood meal.

Factors favouring malaria transmission include environmental factors such as the presence of bushes and stagnant ponds of water around residence, warm climates and rainfall among others. But also, factors related to human behaviour favour malaria parasite transmission [6-10]. These include farming near homes; urban development with poor drainage, dressing that exposes the body to bites of mosquito vector as well as nocturnal activities, given that the vector is nocturnal, biting from dusk to dawn with peaks at about 10 pm. Globally, according to WHO 2023, in 2022, there were an estimated 249 million malaria cases in 85 malaria-endemic countries and areas, an increase of 5 million cases compared with 2021 causing 608000 deaths annually. About 94 % of malaria cases occured in the WHO African Region with 508000 deaths at a rate of 56 per thousand down from 61 per thousand in 2020 [5]. In Cameroon, an average of 6.459.013 cases of malaria and 12.587 deaths were recorded in 2022 [5]. Bamenda in northwest Cameroon is a hypoendemic region for malaria transmission [11]. Studies have reported varied prevalence of malaria in Bamenda among different groups of individuals. Reports by Ngum *et al.* [12] and Ebai *et al.* [13] have shown 26.7% prevalence among patients consulting at the Regional Hospital Bamenda (RHB) and 18.0% among pregnant women attending ANC at the same facility respectively.

The severity of malaria has been associated with the intensity of infected vector bites, and the general health status of the infected person. The parasite, especially *P. falciparum,* is associated with immunosuppression and massive destruction of red blood cells leading to anaemia, asthenia, respiratory difficulties and cerebral malaria among other manifestations. These could be severe sometimes especially in children less than five years of age, pregnant women, non-immune and immunosuppressed individuals including those having underlying diseases like HIV/AIDS. In the latter case, there could be more severe disease condition and increased mortality. However, an efficient management of the comorbidities like malaria is susceptible to averting the severity. Unfortunately, malaria testing is not done as a routine check for HIV patients on treatment in the RHB probably because it is not inscribed in the National Strategic Plan For fight against HIV/AIDS and STIs [14].

HIV/AIDS is an immunosuppressive disease that targets the immune system of the body through the ~~infection~~ and destruction of CD4+ cells ~~which are~~ a clone of T-lymphocytes (white blood cells) that play an important role in immune responses. CD4+ cells help in the activation of B-lymphocytes to plasma cells which secret immunoglobulins responsible for humo~~u~~ral immune responses against infections in the body [15]. HIV transmission is principally through unprotected sexual intercourse, but also through transfusion of infected blood, ~~IV~~ drug use and from mother to child especially during childbirth as well as accidental exposure to infected blood. HIV/AIDS patients often present with reduced immunity due to reduction of CD4+ cells over time~~.~~ ~~This exposes~~ the patients to opportunistic infections by bacteria, fungi, viruses and parasites. This further leads to severe disease that presents with persistent fever, diarrhoea, weight loss and death among others [16]. According to WHO, about 39 million people worldwide live with HIV; 1.3 million new cases were recorded in 2022 with about 630,000 deaths worldwide. Among the people living with HIV (PLHIV) 89% (29.8 million) ~~is~~ receiving antiretroviral therapy (ART) [17]. In the WHO African region, an estimated 25.6 million people lived with HIV in 2022, of which 82% (20.9 million) received ART. An estimated 660 000 people were infected with HIV in 2022 while 380 000 [300 000–540 000] deaths were attributed to HIV-related causes in 2022 [17].

In Cameroon, an estimated 480000 people are infected with HIV, 9900 new cases of infection were recorded in 2023 with about 2.6% (10,000) deaths [18]. A total of about 312,214 people are on ARVs in Cameroon with about 11% of them in the North West Region where the HIV prevalence is 4% [14]. Some 4000-5000 HIV-positive individuals including about 15 newly diagnosed HIV-positive individuals are received at the treatment centre of the RHB each month where treatment is done using ART as prescribed by the national guidelines. Three therapeutic protocols divided into first, second and third lines are available with different combinations of antiretroviral.

Over the years, the approach to care for HIV patients has improved from treating patients based on reduced levels of CD4+ cells in blood and high viral loads in the late 1990s to operation test and treat all positive (option B+) recently [14]. In ~~this~~ option, patients are enrolled to treatment protocols once tested and confirmed positive~~ in a treatment centre also known as day hospital~~. This approach aims to reduce the morbidity and mortality associated with HIV/AIDS. HIV treatment is expected to boost the immunity of patients by inhibiting virus replication in the body and therefore reduce their vulnerability to other infections. That notwithstanding, there are reports on comorbidities like malaria in HIV patients under treatment in Cameroon with varied prevalence. These include 58.9% in Limbe, Southwest of Cameroon ~~by Kimbi *et al.* in 2012~~ [19], 2.24% in Bamenda, Cameroon i~~n 2012 by Njunda *et al.*~~ [20] and 24.5% in Regional Hospital Bamenda ~~in 2018 by Eyong *et al.*~~ [21]. Both diseases contribute to immunosuppression with sometimes exacerbated outcomes [22]. Based on the above, the objectives of this study were to determine the prevalence of malaria parasitaemia, clinical manifestations, factors associated with malaria parasitaemia and the parasite density in HIV patients on ART at the treatment centre of the Regional Hospital Bamenda in northwest of Cameroon. The findings of this study are expected to provide a guide in the conception of an integrated approach to the control of malaria and reduction of morbidity and mortality among HIV/AIDS patients on ART.

2. Materials and Methods:

2.1. Study design

This was a hospital-based cross-sectional study carried out in the HIV treatment centre of the RHB during the months of April ~~2nd~~ and May~~ 31st~~ 2023. The ~~study~~ subjects were HIV~~-~~positive patients on treatment ~~admitted~~ after ~~receiving~~ their informed consent. A pre-tested questionnaire was used to collect data on socio-demographic characteristics, clinical manifestations, and history of HIV treatment as well as use of malaria prevention methods. ~~Capillary blood was collected to prepare thick and thin blood films that were Giemsa-stained~~

and viewed under a light microscope for malaria detection, quantification and identification. A drop of blood was also used to determine white blood cell count.

2.2. Study area and population

The study was carried out at the HIV/AIDS treatment centre of RHB which is the highest referral hospital in the region. It is the largest treatment centre in the North West Region of Cameroon. The centre is known to receive on average 4000-5000 HIV-positive individuals with about 15 newly diagnosed HIV-positive individuals each month. Bamenda has a predominantly savannah type of vegetation just like the majority of the region. It is located at longitude 100.08 to 100.12 E and latitude 50.55 to 60.00 N, and about 1258-1770m above sea level. Bamenda has two seasons, a rainy season that spans from April to November and a dry season from December to March. With an average temperature of 23°C, March is the warmest month while July is the coldest with an average temperature of 20.1°C. The average annual rainfall is 2145 mm [23, 24]. Bamenda is the capital of the North West Region of Cameroon, a cosmopolitan city with several private and state-owned health facilities and educational establishments. Economic activities are represented at small, medium and large scales, however, farming predominates.

The study population was made up of HIV/AIDS-positive individuals of both sexes receiving treatment at the Day Hospital of the RHB who are on one of the following antiretroviral protocols. First-line ART regimens: 7A (Abacavir, lamivudine, Dolukegravir); 8A (Tenofovir, lamivudine, Dolukegravir) and 9A (Tenofovir, lamivudine, Efavirenz). Second-line ART regimens: 10A (Douvir, lamivudine, Dolukegravir); 12A (Douvir, lamivudine, Atazanavir) and third-line ART regimens: 19A (Tenofovir, lamivudine, Dolukegravir). Participants on anti-malarial medication within one week to admission in the study were excluded.

Sampling and Sample Size Estimation

The convenient sampling technique was used. The sample size was computed using the Cochrane formula ($n=Z^2pq/d^2$) [25], where n is the required sample size, Z is 1.96, the standard normal deviation for 95% confidence interval, p was 24.5% is the prevalence of malaria among

HIV patients on treatment reported by Eyong *et al* in a study in Bamenda [21], q=1-p, while d is 0.05 which is the acceptable level of error set for the study. The sample size was calculated at 284 participants. However, just 181 participants met the criteria for recruitment and accepted to be part of the study.

2.3. Data Collection:

2.3.1 Administration of questionnaire

After explaining the study procedure to the participants, a pretested questionnaire was administered to those who consented. Data was collected on socio-demographic characteristics, clinical manifestations, history of ART, environmental factors and the methods of malaria prevention. The questionnaire was administered in English and exceptionally in Pidgin English and French for participants who preferred one of these two Languages.

2.3.2 Laboratory Procedures

Capillary blood sample was collected from the participants by finger prick. 70% alcohol was used to disinfect a tip of the finger before pricking with a sterile lancet to obtain blood. Two drops of blood were collected on a glass slide and used to prepare thick and thin blood smears. The smears were allowed to air-dry. After drying, the thin smears were fixed with absolute methanol. Both thick and thin smears were then stained with 10% Giemsa stain for 10 minutes. Malaria parasitaemia per µl of blood (MP/-µl) was determined by counting malaria parasite stages against 500 white blood cells and the final value obtained from the formula MP= counted parasites/500*WBC count [26]. Up to 500 WBCs were counted per smear because of the very low parasitaemia observed. Each smear was read by two parasitologists using a binocular light microscope and in case of a discrepancy, the value of a third reader was considered. Another drop of blood was collected to determine the white blood cell count (WBC) using the improved Neubauer counting chamber. A 1/20 dilution was prepared using a solution of acetic acid tinged in 1% gentian violet. A drop was mounted on the Neubauer counting chamber and WBCs were counted in the four large squares and WBC count determined using standard formula [26].

2.4. Data Analysis

Data after collection was entered into Microsoft Excel Spread Sheet and exported to the statistical package of social science (SPSS), version 20.0 for analyses. Demographic characteristics such as gender and age were summarized into percentages. The Chi-square test was used to compare proportions while odds ratio (OR) was used to determine risk factors. The

variation in geometric mean parasite densities (GMPD) was determined using one-way Anova test. The level of significance was set at $p < 0.05$.

2.5. Ethical Considerations:

The ethical clearance to carry out this study was obtained from the Institutional Review Board of the University of Bamenda hosted by the Faculty of Health Sciences. An administrative clearance for the study was obtained from the Regional Delegation of Public Health for the North West Region while an authorization was obtained from the RHB administration. Written informed consent was obtained from participants after providing information on the procedures, risk and advantages of the study. Participation in the study was totally free and voluntary. Participants could quit the study at any time without any penalty. Confidentiality was respected by the use of codes on all documentations. All patients diagnosed of malaria parasitaemia were referred to a clinician for treatment.

3. Results

3.1. Socio-demographic characteristics of study participants

A total of 181 participants took part in this study. The age range was 21-71 years with a mean±SD of 46.53±11. The most represented age group was 21-39(26.5%). There were more females 138(76.2%) than males. Majority of the participants 158(87.3%) were resident in Mezam Division of the North West Region. With respect to level of education, 78 (43.1%) of the participants ~~had attained~~ primary. Most of the participants 171 (94.5%) were Christians. (Table1).

Table 1: Socio-demographic characteristics of the study participants

Characteristic	Category	Frequency	Percentage
	Mean ±SD	46.53±11	
	Range	21-74	

**Age group (years)**	21-39	48	26.5
	40-47	47	26.0
	48-54	43	23.8
	55-71	43	23.8
**Sex**	Female	138	76.2
	Male	43	23.8
**Address**	Mezam	158	87.3
	Others	23	12.7
**Level of education**	No formal education	17	9.4
	Primary	78	43.1
	Secondary	68	37.6
	Tertiary	18	9.9
**Religion**	Christian	171	94.5
	None	10	5.6
**Occupation**	Business	57	31.5
	Farmer	54	29.8
	Non salary earners	48	26.5
	Salary earners	22	12.2
**Marital status**	Married	101	55.8

Single	51	28.2
Widowed	29	16.0
Total	181	100.0

3.2. Prevalence of malaria parasite in HIV-positive patients

3.2.1. Prevalence of malaria parasite in HIV patients ~~with respect to~~ sociodemographic characteristics

The overall prevalence of malaria was 9.4% (17/181). Only *Plasmodium falciparum* ~~species~~ was detected. Although there was no significant statistical difference in the prevalence of malaria ~~with respect to~~ sociodemographic characteristics, a higher prevalence was observed amongst individuals aged 55-71 (14%), females (10.1%), those with no formal education (17.6%), ~~business~~ persons (12.3%) and widows (10.3%) compared to their corresponding counterparts. This is shown in Table 2.

Table 2: Prevalence of malaria parasite ~~with respect to~~ socio-demographic characteristics

Characteristics	Category	No. tested (%)	~~Prevalence~~ (%)	χ²(p-value)
**Age (years)**	21-39	48 (26.5)	3 (6.3)	1.99 (0.57)
	40-47	47(26.0)	5 (10.6)	
	48-54	43(23.8)	3 (7.0)	
	55-71	43(23.8)	6 (14.0)	
**Sex**	Female	138(76.2)	14 (10.1)	0.38 (0.53)
	Male	43(23.8)	3 (7.0)	

**Address**	Mezam	158(87.3)	16 (10.1)	0.78 (0.37)
	Others	23(12.7)	1 (4.3)	
**Level of education**	No formal education	17(9.4)	3 (17.6)	3.32 (0.34)
	Primary	78(43.1)	5 (6.4)	
	Secondary	68(37.6)	6 (8.8)	
	Tertiary	18(9.9)	3 (16.7)	
**Religion**	Christian	171(94.5)	16 (~~9.3~~)	0.03 (0.86)
	None	10(5.6)	1 (~~11.1~~)	
**Occupation**	Business	57(31.5)	7 (12.3)	3.1 (0.38)
	Farming	54(29.8)	2 (3.7)	
	Non salary earners	48(26.5)	5 (10.4)	
	Salary earners	22(12.2)	3 (13.6)	
**Marital status**	Married	101(55.8)	9 (8.9)	0.07 (0.97)
	Single	51(28.2)	5 (9.8)	
	Widowed	29(16.0)	3 (10.3)	
	Total	181 (100)	17 (9.4)	

3.2.2. Prevalence of malaria parasite ~~with respect to~~ treatment and prevention methods

Although there was no significant statistical difference in the prevalence of malaria ~~with respect to therapeutic~~ protocols (χ^2=1.03, p=0.96), a higher prevalence was observed in participants who were taking first-line treatment, Tenofovir, Lamivudine, Dolukegravir (8A) (~~10.3~~%, 14/122). Participants with malaria parasitaemia were from 8A and 12A ART which are in the first and second lines of ART respectively. Regarding viral load, the prevalence of malaria was higher in patients with detectable viral load (14.3%, 3/21) than among those with undetectable viral load (~~4.8~~%, 14/160), although the difference was as well not statistically significant (OR=0.57 (CI: 0.15-2.2), p=0.41). With respect to malaria prevention methods reported by the participants, it was observed that participants who do not use mosquito nets (11.3%, 13/115), those who ~~did~~ not use insecticide spray (10.4%, 16/154) and those who closed doors and windows after 5:00pm (12.9%, 12/110) had a higher malaria prevalence than their respective counterparts although none of these showed a significant statistical difference as seen in Table 3.

Table 3: Prevalence of malaria parasite ~~with respect to treatment related factors~~ and prevention methods

**Factor/method**	**Category**	**Nº. tested**	**~~Prevalence~~ (%)**	**Test**	**P-value**
**ARV**	7A	1	0 (0.0)	χ^2=1.03	0.96
	8A	122	14 (~~10.3~~)		
	9A	3	0 (0.0)		
	10A	1	0 (0.0)		

	12A	34	3 (~~8.1~~)		
	19A	3	0 (0.0)		
**Last viral load**	Detectable	21	3 (14.3)	OR=.0.57 0.15-2.2)	0.41
	Undetectable	160	14 (~~4.8~~)		
**Live by bush**	No	66	4 (6.1)	χ^2 =1.36	0.24
	Yes	115	13 (11.3)		
**Live by stagnant water**	No	145	14 (9.7)	χ^2 =0.59	0.89
	Yes	36	3 (8.3)		
**Use mosquito net**	No	68	7 (~~9.3~~)	χ^2 =0.001	0.98
	Yes	96	10 (~~9.4~~)		
**Use insecticide spray**	No	154	16 (10.4)	χ^2 =1.21	0.27
	Yes	27	1 (3.7)		
**Type of house**	Cement	140	14 (10.0)	χ^2 =0.27	0.61
	Mud brick	41	3 (7.3)		
**Close doors and windows**	After 5pm	110	12(10.9)	χ^2 =0.06	0.81
	Before 5pm	71	5(7.0)		

First-line ART: 7A=Abacavir, lamivudine, Dolukegravir; 8A=Tenofovir, lamivudine, Dolukegravir; 9A= Tenofovir, lamivudine, Efavirenz. Second-line ART: 10A=Douvir, lamivudine, Dolukegravir; 12A=Douvir, lamivudine, Atazanavir. Third-line ART: 19A= Tenofovir, lamivudine, Dolukegravir.

3.2.3. Prevalence of malaria parasite ~~with respect to~~ clinical manifestations~~:~~

Comparing the prevalence of malaria parasitemia ~~with respect to presence of~~ clinical manifestations, ~~it was observed that,~~ the prevalence was higher among patients who presented with dyspnea (25%, 3/12) than their counterparts ~~and the difference was statistically significant~~. Those with dyspnoea were ~~at~~ four times more ~~odds~~ to have malaria parasite than ~~their counterparts~~ (OR= 3.7 (0.9-15.2), p=0.04). The prevalence was also higher in participants with fever (14.7%, 5/34) who were ~~at~~ two times more ~~odds of having~~ malaria parasite (OR=1.94 (CI: 0.63-5.93), p=0.24)~~,~~ chills (13.3%, 4/30) who were ~~at~~ two times more ~~odds~~ (1.63 (CI: 0.50-5.40), p=0.42) to be infected by malaria parasite as well as headache (12.3%, 8/65), who were also ~~at~~ 2 times more ~~odds of having~~ malaria parasite (1.70 (CI: 0.61-4.6), p=0.31) than their corresponding counterparts ~~although there was no significant statistical difference as seen in~~ Table 4.

Table 4: Malaria parasite ~~with respect to~~ clinical manifestations

Clinical manifestation	Category	No. tested	~~Prevalence~~	OR (95% CI)	p-value
**Fever**	No	147	12 (8.2)	1.94 (0.63-5.93)	0.24
	Yes	34	5 (14.7)		
**Chills**	No	151	13 (8.6)	1.63 (0.50-5.40)	0.42
	Yes	30	4 (13.3)		
**Abdominal pain**	No	151	14 (9.3)	1.10 (0.29-4.04)	0.90
	Yes	30	3 (10.0)		

**Headache**	No	116	9 (7.80)	1.70 (0.61-4.6)	0.31
	Yes	65	8 (12.3)		
**Nausea**	No	167	14 (8.4)	3.0 (0.74 - 11.95)	0.11
	Yes	14	3 (21.4)		
**Diarrhea**	No	157	14 (8.9)	1.50 (0.39 - 5.50)	0.57
	Yes	24	3 (12.5)		
**Dyspnea**	No	169	14 (8.3)	3.70 (0.90 - 15.2)	0.04
	Yes	12	3 (25.0)		
**Fatigue**	No	113	8 (7.1)	2.0 (0.73 – 5.50)	0.17
	Yes	68	9 (13.2)		
**Joint/muscle pain**	No	134	10 (7.5)	2.17 (0.77 – 6.07)	0.13
	Yes	47	7 (14.9)		

3.2.4. Variation in malaria parasite density (GMPD/µl of blood) ~~with treatment-related factors~~

Comparing the GMPD of participants with respect to treatment-related factors using one-way Anova test, showed no significant statistical difference. However, there was a general tendency for GMPD to increase with duration of treatment. Patients who had been on treatment for at least 4 years had the highest GMPD (50±20 parasites/µl). Only patients on the 8A and 12A therapeutic protocols were infected with malaria parasite with GMPD of 48.6±17.5 and 40±00

per µl of blood respectively. There was also a tendency for higher GMPD among participants with detectable viral load (53.3±23.1 parasites~~-~~/µl of blood) than those with undetectable viral load (45.7±14.5 parasites~~-~~/µl of blood). ~~This is shown in~~ Table 5.

Table 5: Variation in GMPD ~~with treatment related factors~~

Factor	Category (n)	GMPD (±SD)	F	p-value
Duration of treatment (years)	1(2)	40.0 (0.0))	0.3	0.59
	2(5)	48.0 (17.9)		
	3 (6)	46.7 (16.3)		
	4 (4)	50.0 (20.0)		
Treatment type	8A (14)	48.6 (17.5)	0.72	0.41
	12A (3)	40.0 (0.0)		
Viral load	Detectable (3)	53.3 (23.1)	0.56	0.46
	Undetectable (14)	45.7 (14.5)		

4. Discussion

Malaria and HIV/AIDS co-infections continue to occur despite the measures put in place by governments to fight against these diseases which are endemic in sub-Saharan Africa including Cameroon. ~~However,~~ these measures are yielding remarkable results as can be seen with the relatively low prevalence and low parasite densities recorded in this study. An overall prevalence of 9.4% ~~malaria parasitaemia~~ was observed in this study~~.~~ ~~This is~~ lower than the 58.9% reported by Kimbi *et al.* [19] and 24.5% by Eyong *et al.* [21], ~~all of which were reported over a decade ago~~ among HIV/AIDS patients on ART in Cameroon~~.~~ ~~The prevalence was also lower than~~ 23.7% reported by Wondimeneh *et al.* in Ethiopia [28]. There has been a progressive

decrease in malaria prevalence on the global and national scales due to a scale-up ~~in the~~ measures to combat the disease. ~~Also~~, in the past, ART was not free ~~and on the other hand,~~ there has been a lot of education ~~on~~ mass media especially in hospital settings. This might have benefitted the masses especially HIV patients receiving treatment. However, an upsurge in prevalence was observed in 2020 due to a breakdown in health systems ~~by~~ the Covid 19 pandemic [17]. The prevalence of malaria parasitemia in this study was higher compared to 2.2% reported by Njunda *et al.* in 2016 among PLWHIV in Yaounde, the national capital of Cameroon [20]. The effective implementation of ART started in Yaounde before decentralization to treatment centers in the regions and districts. It is natural that with this, the Yaounde patients on treatment would have been better protected against other infections including malaria. The prevalence of malaria parasitemia in this study was also higher than 7.8% reported by Akinbo *et al.* in 2016 in Kogi state, Nigeria [27]. The higher prevalence observed ~~here~~ could be due to variability in the dynamics of malaria transmission in different ecological settings as well as variations in the strengths of health systems.

Regarding sociodemographic characteristics, the absence of significant variation in prevalence of malaria among the participants in this study is similar to ~~other~~ findings. Sahle *et al.* in Ethiopia [29] and Simon-oke *et al.* in Nigeria [30] ~~reported that~~ there was no significant statistical difference between age groups among participants in similar studies. ~~In the same light,~~ Kwenti *et al.* [31] in a study in Yaounde, Cameroon and Gumel *et al.* in northwest Nigeria [32] reported ~~the absence of a~~ significant statistical difference ~~between~~ malaria parasite prevalence between gender and occupation respectively. There is likelihood that the ART received placed participants in ~~a~~ similar state in terms of defence against infections. Furthermore, there could be a general sense of awareness of the precarious state of ~~the~~ health ~~of the patients~~ that ~~urges~~ them to adopt similar healthy behaviours. Given that ART is prescribed following ~~the~~ particularities ~~of each individual~~, sociodemographics may not play a significant role on the vulnerability of the patient to malaria. However, the study by Akinbo *et al.* in Nigeria [27], reported a significant statistical difference in prevalence with respect to gender.

This study showed that there were no significant statistical differences in malaria prevalence and density (GMPD) between ART protocols as well as level of viral load. There is a possibility of similarity in immune status provided by the ART to patients and the implementation of malaria prevention methods. Similar findings were reported by Tchinda *et al.* in Douala, Cameroon [33], Jegede *et al.* in Nigeria [34] and Ejigu *et al.* in southwest Ethiopia [35]. The detection of malaria parasitemia among patients on 8A and 12A ART protocols may have been due to the fact that the majority of the participants were enrolled into these two. Lower prevalence and density of parasitemia among patients with undetectable viral load could be an indication of improved immunity.

It was observed that there is no significant statistical difference in the prevalence of malaria with respect to malaria prevention methods. There was however a tendency for malaria parasite prevalence to be lower among those who closed their doors and windows before 5pm, those who used bed nets, and those who used insecticidal sprays. These measures are evidently protective against malaria transmission. Studies have shown varied reports on this, with some showing no significant statistical difference [Jegede *et al* [34] while others have reported significant statistical difference [Akinbo *et al* [27]. This can be due to peculiarities in study populations and other factors including level of endemicity of malaria in the different areas.

The higher malaria parasitemia observed among patients with dyspnea is an indication that malaria is associated with difficulty in respiration. This could be due to an increased respiratory rate to compromise low levels of haemoglobin, which is associated with *Plasmodium falciparum* infection as a result of massive destruction of red blood cells. Furthermore, although there was no significant statistical difference in malaria parasite prevalence with respect to the other clinical manifestations, the signs and symptoms the patients presented may have been due the HIV infection, but also the type of activity that they carry-out, given that most of them were business persons, farmers and non-salary earners, these are generally associated with some physical activity that may contribute to fatigue, pains, headache and other manifestations.

~~It should be noted that, no confirmation of malaria parasitemia using PCR was done apart from microscopy. This~~ might have limited the chances of detecting parasitemia in patients with lower densities. Also, data ~~collected~~ on clinical manifestations were based on ~~patients~~' responses, ~~so~~ the results ~~may have been affected by the subjectivity of the participants. The~~ sample size was not attained due to patients' refusal to participate.

5. Conclusion

~~From the results obtained in this study, it can be concluded~~ that there are low levels of malaria parasite prevalence and density amongst HIV patients on ART in the treatment center of the Bamenda Regional Hospital. Apart from dyspnea as a clinical manifestation, the prevalence of malaria parasitemia and parasite density ~~was~~ similar among patients although tendencies of variation in manifestations were observed between some groups. A systematic malaria test could be helpful in averting morbidity and improving on the general health of patients on ART.

Acknowledgements

The authors acknowledge the authorities of the Regional Hospital Bamenda for accepting that this study should be carried out in the facility. We also acknowledge the participants for accepting to take part in the study.

References

1. World Health Organization. HIV country intelligence. ~~Cameroon~~ HIV country profile 2023. https://cfs.hivci.org/index.html
2. Antinori S, Galimberti L, Milazzo L, Corbellino M. Plasmodium knowlesi: the emerging zoonotic malaria parasite. Acta Trop. 2013;125:191–201.
3. White NJ. Anaemia and malaria. Malar J. 2018;17:371. Available:https://doi.org/10.1186/s12936- 018-2509-9
4. Jamison DT, Feacham RG, Makgoba MW, Bos ER, Baingana FK, Hofman KJ et al. Disease and Mortality in Sub-Saharan Africa, Second Edition. © Washington, DC: World Bank; 2006. Available at: http://hdl.handle.net/10986/7050 License: CC BY 3.0 IGO."

5. World malaria report 2023. Geneva: World Health Organization; 2023. Licence: CC BY-NC-SA 3.0 IGO.
6. Musoke D, Miiro G, Ndejjo R, Karani G, Morris K, Kasasa S, Nakiyingi-Miiro J, Guwatudde D, Musoke MB. Malaria prevention practices and associated environmental risk factors in a rural community in Wakiso district, Uganda. PLoS One. 2018 Oct 9;13(10):e0205210. doi: 10.1371/journal.pone.0205210. PMID: 30300396; PMCID: PMC6177175.
7. Kibret S, Glenn-Wilson G, Ryder D, Tekie H, and Petros B. Environmental and meteorological factors linked to malaria transmission around large dams at three ecological settings in Ethiopia. *Malar J* 18, 54 (2019). https://doi.org/10.1186/s12936-019-2689-y
8. Rouamba T, Nakanabo-Diallo S, Derra K, Rouamba E, Kazienga A, Inoue, Y, Ouédraogo EK, Waongo M, Dieng S, Guindo A, Ouédraogo B, Sallah KL, Barro S, Yaka P, Kirakoya-Samadoulougou F, Tinto H and Gaudart J. Socioeconomic and environmental factors associated with malaria hotspots in the Nanoro demographic surveillance area, Burkina Faso. BMC Public Health (2019) 19:249 https://doi.org/10.1186/s12889-019-6565-z
9. Nyasa RB, Fotabe EL, Ndip RN. Trends in malaria prevalence and risk factors associated with the disease in Nkongho-mbeng; a typical rural setting in the equatorial rainforest of the South West Region of Cameroon. PLoS One. 2021 May 18;16(5):e0251380. doi: 10.1371/journal.pone.0251380. PMID: 34003847; PMCID: PMC8130964.
10. Kimbi HK, Nana Y, Sumbele IUN, Anchang-Kimbi JK, Lum E, Tonga C, Nweboh M and Lehman LG. Environmental Factors and Preventive Methods against Malaria Parasite Prevalence in Rural Bomaka and Urban Molyko, Southwest Cameroon. J Bacteriol Parasitol 2013, 4:1 http://dx.doi.org/10.4172/2155-9597.1000162.
11. Same-Ekobo A, Fondjo E, Eouzan JP. Grands travaux et maladies à vecteurs au Cameroun Impact des aménagements ruraux et urbains sur le paludisme et autres maladies à vecteurs., Paris: IRD Ed, Collection expertise collégiale; 2001.

12. Ngum NH, Fakeh NB, Lem AE ~~*et al*~~. Prevalence of malaria and associated clinical manifestations and myeloperoxidase amongst populations living in different altitudes of Mezam division, North West Region, Cameroon. *Malar J* 22, 20 (2023). https://doi.org/10.1186/s12936-022-04438-6

13. Ebai CB, Ebongue FNE, Lum OKT, Musoro JZ, Yamssi C and Kimbi HK. Plasmodium falciparum Parasitaemia during Pregnancy and the Use of Malaria Prevention Methods by Women Attending Antenatal Consultation at the Regional Hospital Bamenda, Northwest Cameroon. International Journal of Tropical Disease & Health, 2020; 41(20):46–56. Available at: https://doi.org/10.9734/ijtdh/2020/v41i2030393

14. Cameroon National Strategic Plan For fight against HIV/AIDS and STIs 2021-2023. Ministry of Public Health, Cameroon.

15. Luckheeram RV, Zhou R, Verma AD, Xia B. CD4^{+}T cells: differentiation and functions. Clin Dev Immunol. 2012;2012:925135. doi: 10.1155/2012/925135. Epub 2012 Mar 14. PMID: 22474485; PMCID: PMC3312336.

16. Institute of Medicine ~~(US) Committee on a National Strategy for AIDS~~. Confronting AIDS: Directions for Public Health, Health Care, and Research. Washington ~~(DC)~~: National Academies Press ~~(US)~~; 1986. ~~A, Clinical Manifestations of HIV Infection.~~ Available from: ~~https://www.ncbi.nlm.nih.gov/books/NBK219125/~~

17. World Health Organization HIV statistics, globally and by WHO region ~~Epidemiological factsheet.~~ Global HIV, Hepatitis and Sexually Transmitted Infections Programmes ~~20, avenue Appia 1211~~ Geneva ~~27~~ Switzerland. ~~who.int.~~ Available from: https://www.who.int/teams/global-hiv-hepatitis-and-stis-programmes/hiv/strategic-information/hiv-data-and-statistics

18. World Health Organization 2023 data.who.int, Cameroon [Country overview]. (Accessed on 28 December 2023).

19. Kimbi HK, Njoh DT, Kenneth JN Ndamukong KJN and Lehman LG. Malaria in HIV/AIDS Patients at Different CD4+ T Cell Levels in Limbe, Cameroon. Journal of Bacteriology & Parasitology 2012, 04. DOI: 10.4172/2155-9597.1000164.

20. Njunda AL, KamgaHL, Nsagha DS, Assob JCN, Kwenti TE. Low Malaria Prevalence in HIV-Positive Patients in Bamenda, Cameroon. Journal of Microbiology Research 2012, 2(3): 56-59 DOI: 10.5923/j.microbiology.20120203.03

21. Eyong A, Kechia F, Tembe E, Asaah TC, Wandum C, Ashu A, Mbanya D, Fokunang T, Gangdia C, Ashu M, Shu D, & Fokunang, C. Evaluation of Malaria Parasitemia Among HIV/AIDS Individuals Attending the Bamenda Regional Hospital Treatment Center. Central African Journal of Public Health 2019. 5. 65-76. DOI:10.11648/j.cajph.20190502.12.

22. Mahittikorn A, Kotepui KU, De Jesus MG, Masangkay FR, Kotepui M. A meta-analysis on the prevalence and characteristics of severe malaria in patients with Plasmodium spp. and HIV co-infection. Sci Rep. 2021 Aug 17;11(1):1–19. DOI:10.1038/s41598-021-95591-6Corpus ID: 237198069.

23. Available:https://weather-and-climate.com/average-monthly-Rainfall-Temperature-Sunshine,bamenda-cm,Cameroon.

24. Available:https://en.climate-data.org/africa/cameroon/northwest/bamenda-2905/

25. Cochrane WG. Sampling Techniques, Wiley, New York; 1963.

26. Cheesbrough M. Laboratory Practice in Tropical Countries part 1. Cambridge University Press. 2006;2(4):191-235.

27. Akinbo F, Anate P, Akinbo D, Omoregie R, Okoosi S, Abdulsalami A, et al. Prevalence of malaria among HIV patients on highly active antiretroviral therapy in Kogi State, North Central Nigeria. Ann Niger Med. 2016;10(1):11. DOI:10.4103/0331-3131.189802Corpus ID: 78792478.

28. Wondimeneh Y, Gebrecherkos T, Muluye D, Damtie D, Ferede G. HIV and Malaria Infections and Associated Risk Factors Among Febrile Illness Patients in Northwest

Ethiopia. Turkiye Parazitol Derg. 2018 Sep;42(3):180-186. DOI: 10.5152/tpd.2018.5878. PMID: 30109850.

29. Sahle T, Yemane T, Gedefaw L. Effect of malaria infection on hematological profiles of people living with human immunodeficiency virus in Gambella, southwest Ethiopia. BMC Hematol. 2017 Dec;17~~(1):1–8~~. https://doi.org/10.1186/s12878-017-0072-1.

30. Simon-Oke IA, Ade-Alao AO, Ologundudu F. The impact of HIV-associated immunosuppression on the Plasmodium falciparum chloroquine resistance transporter gene (PfCRT) of HIV patients in Akure, Nigeria. Bull Natl Res Cent. 2020 Dec;44~~(1):1–7~~. DOI:10.1186/s42269-020-00401-0~~Corpus ID: 221778446~~.

31. Kwenti TE, Edo E, Ayuk BS, Kwenti TDB. Prevalence of Coinfection with Malaria and HIV among Children in Yaoundé, Cameroon: A Cross-Sectional Survey Performed in Three Communities in Yaoundé. Yangtze Med. 2017 Sep 20;1(3):178–88. DOI: 10.4236/ym.2017.13018.

32. Gumel SD, Ibrahim A, Olayinka AT, Ibrahim MS, Balogun MS, Dahiru A, et al. HIV-malaria co-infection and its determinants among patients attending antiretroviral treatment clinic in Zaria, ~~North-Western~~, Nigeria. ~~bioRxiv~~. ~~2019 Mar 25;588855~~. DOI: https://doi.org/10.37432/jieph.2021.4.1.31.

33. Tchinda GG, Atashili J, Achidi EA, Kamga HL, Njunda AL, Ndumbe PM. Impact of Malaria on Hematological Parameters in People Living with HIV/AIDS Attending the Laquintinie Hospital in Douala, Cameroon. PLoS ONE ~~[Internet]~~. 2012 ~~[cited 2023 Jun 26]~~;7(7). ~~Available from: https://www.ncbi.nlm.nih.gov/pmc/articles/PMC3393653/. https://doi.org/10.1371/journal.pone.0040553.~~

34. Jegede FE, Oyeyi TI, Abdulrahman SA, Mbah HA, Badru T, Agbakwuru C, et al. Effect of HIV and malaria parasites co-infection on immune-hematological profiles among patients attending anti-retroviral treatment (ART) clinic in Infectious Disease Hospital

Kano, Nigeria. PLOS ONE. 2017 Mar 27;12(3):e0174233. https://doi.org/10.1371/journal.pone.0174233.

35. Ejigu S, Haile D, Solomon Y. Effect of malaria and HIV/AIDS co-infection on red blood cell indices and its relation with the CD4 level of patients on HAART in Bench Sheko Zone, Southwest Ethiopia. PLOS ONE. 2022 Mar 4;17(3):e0263865. https://doi.org/10.1371/journal.pone.0263865.
