# Supplementary material for: Low levels of Plasmodium falciparum parasitaemia among patients receiving antiretroviral therapy at the treatment centre of the Regional Hospital Bamenda, Northwest Cameroon
Source: PLoS One. 2025 Jan 29;20(1):e0317120. doi: 10.1371/journal.pone.0317120 (PMC11778629; doi:10.1371/journal.pone.0317120)
Supplement: Attachment — Submitted filename: Reviewers Comments.pdf [file pone.0317120.s004.pdf]

Please find below, detailed comments on the **clean copy of the revised manuscript.**

General:

- The English language quality (including punctuations, etc.) just average and needs thorough editing and improvement. There is still a need for **consistency in spellings** throughout the manuscript; examples include dyspnea/dyspnoea; diarrhea/diarrhoea; parasitemia/parasitaemia at different points in the manuscript. These were pointed out in the earlier review.
- There is a mix-up of in-text citations and corresponding references, some of which are listed below. Hence, there is a need for a **painstaking audit and reconciliation of all in-text citations and corresponding references to ensure correctness.**

Abstract:

Line 15: ART … is undefined. It would be necessary to have this acronym preceded by its full meaning while ART is placed in parentheses, i.e., "**Antiretroviral therapy (ART)**". The acronym suffices, subsequently.

Introduction:

- There is a need to underpin the statement in lines 67-70: "***The parasite, especially P. falciparum, is associated with immunosuppression and massive destruction of red blood cells leading to anaemia, asthenia, respiratory difficulties and cerebral malaria, in case parasites are sequestrated in deep veins of the brain, among other manifestations.***" with relevant citation(s)/reference(s) as suggested in the earlier review.
- Line 72: Replace "for which" with "**in whom**"
- Lines 73-74: Recast as "**However, an efficient management of the comorbidities is key to averting this severity.**"
- Lines 75-77: The statement preceding the in-text citation [15] does not align with the citation. The statement is underpinned more by [**14**] (please refer to lines **379-380** under References). The authors need to reconcile this.
- Lines 81-83: The statement "***HIV transmission is principally through unprotected sexual intercourse, but also through transfusion of infected blood, intravenous (IV) drug use and from mother to child, especially during childbirth, as well as accidental exposure to infected blood [17].***") does not align with the citation [**17**]. The publication [**i.e., 17**] was on CD4+ and nowhere in the article was reference made to HIV transmission.
- Lines 94-95: The statement "***In Cameroon, an estimated 480 000 people are infected with HIV, 9, 900 new cases of infection were recorded in 2023 with about 2.6% (12,480) deaths [21].***" The citation may need to be reconciled with both [**21**] and [**22**] under **References** for correctness.
- Line 197: [**15**] is a wrong citation for the preceding statement which aligns more with [**14**] under **References** (please refer to lines **379-380**).
- Line 104: [**15**] is a wrong citation for the preceding statement which is on HIV. [**15**] is a study on malaria!
- Lines 110-111: [**19**], [**20**], and [**21**] are wrong citations. The sentence in these lines align more with [**23**], [**24**] and [**25**], respectively. Please double-check for correctness.

Materials and Methods

Line 135: The in-text citations [26,27] do not align with the preceding statement but more with [**27,28**] (please refer to **References**). Please reconcile/double-check.

Lines 147-148: [25] does not align with Cochran formula which can be found in [**29**] under **References.**

Line 150: [21] does not align with any study in Bamenda by Eyong et al. This citation, i.e., [**21**] is a World Health Organization publication.

Lines 154-157: Recast the sentence to "***After explaining the study objectives and procedures to eligible participants, a pretested questionnaire was administered to those who consented in order to obtain data on socio-demographic characteristics, clinical manifestations, history of ART, environmental factors and the methods of malaria prevention.***"

Line 162: … to disinfect the tip of the finger before pricking. **Which of the fingers? The authors need to state this.**

Lines 168 and 175: [29] does not align with the preceding statement. This citation, i.e., [29] is "Cochran WG. Sampling Techniques, John Wiley and Sons Inc., New York; 1963."

Line 171: delete "**)**"

Results

Table 1: Under religion, None, the 5.6 should be **5.5**. Please note that 10/181 = **5.52486.** This was pointed out in the earlier review.

Table 2: Under χ^2 (p-value) for **Type of house**, amend χ^2 =0.27 to **0.27 (0.61)**.

Discussion

Lines 265-266: [**19**] and [**21**] do not align with Kimbi *et al*. and Achere *et al*., respectively, as stated. They are both WHO publications, while Kimbi *et al*. and Achere *et al*. are placed as [**23**] and [**25**], respectively (please refer to **References**).

Lines 268-269: Please recast to "***combat the disease [31]. Moreover, in the past, ART was not free while there has been a lot of education through the mass media, especially in hospital settings. This might have benefited the masses***".

Line 271: Please recast to "**2020 due to a breakdown in health systems as a result of the Covid 19 pandemic [???].**"

Line 271: [**17**] does not align with the preceding statement.

Line 273: [**20**] is not Njunda *et al*. in 2012, in **References**. It is actually **[24]**.

Line 275: [**30**] is not Akinbo *et al*. in 2016, in **References**. It is actually **[31]**.

Line 280: [**33**] and [**34**] are not Sahle *et al*. and Simon-Oke *et al*. respectively, in **References**. They are actually **[34]** and **[35]**, respectively.

Ditto, all in-text citations and corresponding references onward. The authors need to do a comprehensive review of the in-text citations so as to correspond with their listing in **References**.

Lines 305-307: Please recast to: "***Studies have shown varied reports on this; Jegede et al. [39] showed no significant statistical difference while Akinbo et al. [31] reported significant statistical difference.***"

Line 316: Please amend as follows: "***and non-salary earnings which are generally associated with some physical activity that may contribute***"

References

The correct citations are as follows:

1. Global Health Estimates **2020**: Deaths by Cause, Age, Sex, by Country and by Region, 2000-2019. Geneva, World Health Organization; 2020.

37. Gumel SD, Ibrahim A, Olayinka AT, Ibrahim MS, Balogun MS, Dahiru A, et al. HIV-malaria 450 co-infection and its determinants among patients attending antiretroviral treatment 451 clinic in Zaria, **Kaduna State,** Nigeria. **Journal of Interventional Epidemiology and Public Health, 2021; 4: 2. DOI: https://doi.org/10.37432/jieph.2021.4.1.31.**

A click at the above DOI shows the publication titled as above. The DOI link does not correspond to the citation provided by the authors. The authors may wish to access the link for correctness.

38. Tchinda GG, Atashili J, Achidi EA, Kamga HL, Njunda AL, Ndumbe PM. Impact of Malaria on Hematological Parameters in People Living with HIV/AIDS Attending the Laquintinie Hospital in Douala, Cameroon. PLoS ONE, 2012; 7(7): **e40553. https://doi.org/10.1371/journal.pone.0040553.**

A visit to the link provided in the citation by the authors (i.e., Available from: https://www.ncbi.nlm.nih.gov/pmc/articles/PMC3393653/https://doi.org/10.1371/journal.pone.0040553)., shows that the proper citation is as stated above.

- Line 392: Amend manifestation to **manifestations.**
- Line 394: Amend https://doi.org/10.172226/938. to **https://doi.org/10.17226/938.**
- Line 421: the correct citation is: **Sci Rep. 2021, 11: 16655. DOI:10.1038/s41598-021-95591-6.**
- Lines 446-447: the correct citation is …. **44: 156. DOI:10.1186/s42269-020-00401-0.**
- Lines 464 and 468: Amend PLOS to **PLoS**
