# Supplementary material for: Low levels of Plasmodium falciparum parasitaemia among patients receiving antiretroviral therapy at the treatment centre of the Regional Hospital Bamenda, Northwest Cameroon
Source: PLoS One. 2025 Jan 29;20(1):e0317120. doi: 10.1371/journal.pone.0317120 (PMC11778629; doi:10.1371/journal.pone.0317120)
Supplement: Attachment — Submitted filename: Response to reviewers.docx [file pone.0317120.s003.docx]

Dear Dr. CALVIN EBAI,

Thank you for submitting your manuscript to PLOS ONE. After careful consideration, we feel that it has merit, but in its present form, the manuscript is not suitable for publication in PLOS ONE until after a major revision addressing critical comments raised by the reviewers. Therefore, we invite you to submit a revised version of the manuscript that addresses the points raised during the review process.

Please submit your revised manuscript by May 23 2024 11:59PM. If you will need more time than this to complete your revisions, please reply to this message or contact the journal office at [plosone@plos.org](mailto:plosone@plos.org). When you're ready to submit your revision, log on to https://www.editorialmanager.com/pone/ and select the 'Submissions Needing Revision' folder to locate your manuscript file.

Please include the following items when submitting your revised manuscript:

- A rebuttal letter that responds to each point raised by the academic editor and reviewer(s). You should upload this letter as a separate file labeled 'Response to Reviewers'.
- A marked-up copy of your manuscript that highlights changes made to the original version. You should upload this as a separate file labeled 'Revised Manuscript with Track Changes'.
- An unmarked version of your revised paper without tracked changes. You should upload this as a separate file labeled 'Manuscript'.

If you would like to make changes to your financial disclosure, please include your updated statement in your cover letter. Guidelines for resubmitting your figure files are available below the reviewer comments at the end of this letter.

If applicable, we recommend that you deposit your laboratory protocols in protocols.io to enhance the reproducibility of your results. Protocols.io assigns your protocol its own identifier (DOI) so that it can be cited independently in the future. For instructions see: <https://journals.plos.org/plosone/s/submission-guidelines#loc-laboratory-protocols>. Additionally, PLOS ONE offers an option for publishing peer-reviewed Lab Protocol articles, which describe protocols hosted on protocols.io. Read more information on sharing protocols at <https://plos.org/protocols?utm_medium=editorial-email&utm_source=authorletters&utm_campaign=protocols>.

We look forward to receiving your revised manuscript.

Kind regards,

Segun Isaac OYEDEJI, Ph.D
Academic Editor
PLOS ONE

Journal Requirements:

When submitting your revision, we need you to address these additional requirements.

1. Please ensure that your manuscript meets PLOS ONE's style requirements, including those for file naming. The PLOS ONE style templates can be found at 
https://journals.plos.org/plosone/s/file?id=wjVg/PLOSOne_formatting_sample_main_body.pdf and 
https://journals.plos.org/plosone/s/file?id=ba62/PLOSOne_formatting_sample_title_authors_affiliations.pdf

2. Please amend either the abstract on the online submission form (via Edit Submission) or the abstract in the manuscript so that they are identical.

3. Please include your full ethics statement in the ‘Methods’ section of your manuscript file. In your statement, please include the full name of the IRB or ethics committee who approved or waived your study, as well as whether or not you obtained informed written or verbal consent. If consent was waived for your study, please include this information in your statement as well.

**Additional Editor Comments**:

In addition to the Reviewers' comments outlined below, please attend to the following:

ABSTRACT
Page 1: Lines 24 and 25

Please replace the sentence "Capillary blood was used for malaria parasite diagnosis and to determine white blood cell count" with the actual method that was used for malaria parasite diagnosis, which in this case, is microscopy.

Page 2: Lines 35 and 36

Please remove the sentence "The prevalence of malaria parasitaemia and parasite density was similar despite variations among the patients." from conclusion. You may wish to incorporate it in the Results section.

INTRODUCTION

Page 2: Line 41

The reference [1], cited in support of the statement is inadequate. Please verify.

Page 3: Line 74

Please replace "susceptible" with another term, such as akin, tantamount, etc., to appropriately convey the message.

Page 4: Lines 79 and 80

Please delete the parenthesis (white blood cells). At this level, that knowledge is implied.

RESULTS

In TABLE 1 (Page 10), and Page 9: Line 204, why did the authors provide only two categories for religion: Christianity and None? What about the other forms of religion? I will advise that, if possible, this section of the data should be removed, because only one type of religion was involved and could be regarded as biased.

DISCUSSION

Page 17: Line 272

Please replace "these measures" with "the measures" since they are not listed contextually.

**Reviewers' comments**:

Reviewer's Responses to Questions

**Comments to the Author**

1. Is the manuscript technically sound, and do the data support the conclusions?

The manuscript must describe a technically sound piece of scientific research with data that supports the conclusions. Experiments must have been conducted rigorously, with appropriate controls, replication, and sample sizes. The conclusions must be drawn appropriately based on the data presented.

Reviewer #1: Yes

Reviewer #2: Partly

2. Has the statistical analysis been performed appropriately and rigorously?

Reviewer #1: Yes

Reviewer #2: No

3. Have the authors made all data underlying the findings in their manuscript fully available?

The [PLOS Data policy](http://www.plosone.org/static/policies.action#sharing) requires authors to make all data underlying the findings described in their manuscript fully available without restriction, with rare exception (please refer to the Data Availability Statement in the manuscript PDF file). The data should be provided as part of the manuscript or its supporting information, or deposited to a public repository. For example, in addition to summary statistics, the data points behind means, medians and variance measures should be available. If there are restrictions on publicly sharing data—e.g. participant privacy or use of data from a third party—those must be specified.

Reviewer #1: Yes

Reviewer #2: No

4. Is the manuscript presented in an intelligible fashion and written in standard English?

PLOS ONE does not copyedit accepted manuscripts, so the language in submitted articles must be clear, correct, and unambiguous. Any typographical or grammatical errors should be corrected at revision, so please note any specific errors here.

Reviewer #1: Yes

Reviewer #2: No

5. Review Comments to the Author

Please use the space provided to explain your answers to the questions above. You may also include additional comments for the author, including concerns about dual publication, research ethics, or publication ethics. (Please upload your review as an attachment if it exceeds 20,000 characters)

Reviewer #1: Introduction:
L53 -56: Authors should add migrant and agricultural workers, these are two factors that also contribute to transmission of malaria.

L67: “The severity …..associated with infected vector bites” What do the authors mean by this?

L70: Cerebral malaria is usually the resultant effect of parasite sequestration in into the deep veins and their ability to cross the BBB. Authors should please correct.


Results:

Prevalence of malaria parasites in HIV positive patients. I am still trying to understand why despite the huge difference between malaria prevalence in Female V Male, Mezeam V Others, Christian V None, there is no statistical significance. The authors should please check these calculations again. –Verified ok. None of the results showed a statistically significant difference

Discussion
The discussion should be structured in the way that the results were presented. First, following the general overview of the demographics of the study participants, the authors should discuss how the prevalence of malaria varies in each group: Females/Males, Mezeam/Other residents, Christian/Others etc. And also contextualised the reasons for the observed differences, for instance, is there any biological or behavioural reasons responsible for the observed prevalence in females and males, this should be done also with others.

Very correct! Actually, you can see that the discussion is structured in same way as results. We started with the discussion of the overall results then with respect to socio-demographice characteristics. However, among all the socio-demographic characteristics, none showed significant statistical difference, so we chose to discuss some including age and sex based on the trends observed.

For future studies, the authors should incorporate PCR method of malaria testing as Cameroon is also known for the transmission of non-falciparum malaria.

Agreed

Is there any contra-indication of ACTs with any f the drugs used for treating PLHIV? If not, the authors should also add to their recommendation the need to test and treat PLHIV for malaria when they visit hospitals.

This is important. Although there are reports on the safety of ACT in patients on ARTs, we cannot claim to be exhaustive in our literature search on this issue. So, we decided not to recommend test and treat for PLHIV.

Reviewer #2: The authors are referred to the attached reviewed manuscript (please see track changes) for my detailed comments on all sections of the manuscript. However, please find below, a few of the specific comments/concerns and suggestions:

• The conceptualization of the study was adequate but the execution was inadequate. The manuscript needs to be thoroughly revised to make for a better article. Please see attached "Reviewed" manuscript.
• The authors’ recourse to spelling of the word ‘’Parasitemia’’ interchangeably with ‘’Parasitaemia’’ in the manuscript is not ideal. For consistency and depending on Journal style, the authors should adopt only one spelling; American (parasitemia) or British (parasitaemia).

Noted. That has been corrected.

• Line 41: Reference [1] is exclusive to the assertion on HIV as a leading cause of mortality. The authors need to back up a similar assertion on malaria with a reference.

True. The appropriate reference has been inserted

• Line 61: The authors should double-check the highlighted figures/estimates (6.459.013 and 12.587) for correctness. Could be typos.

Those were typos

• Lines 74/75: The highlighted sentence is vague and needs to be recast for better comprehension.

The word “susceptible” has been replaced by “akin” for comprehension

• Line 90: The authors should please reconcile these estimates 89% (29.8 million).

Agreed! Corrected

• The objectives of the study were clearly stated and the study design described. However, the clinical manifestations and factors associated with malaria parasitaemia among HIV patients on ART, as described in the study, were not appropriate/adequate to address the stated objectives.

The objectives have been reviewed to suit the factors that were measured. “factors associated” has been replaced with “treatment related factors and prevention measures”

Moreover,
• Line 168/169: Authors need to be more specific. They did not state whether or not the two drops of blood for preparation of blood smears were inclusive/exclusive of the very first drop of blood after pricking the finger with a sterile lancet?

The two drops of blood were exclusive of the first drop after pricking the finger with a sterile lancet.

• Line 171: ‘’Both thick and thin films were stained with 10% Giemsa stain …..’’

Reviewed.

• Although this yields faster results, the films may be of less predictable quality than if stained with 2.5% or 3% Giemsa stain. One would have expected that for such a study, either of the latter concentrations would have been used.

Agreed. This is considered a limit of this study.

• Actual sample size (181) was far less than the estimated sample size (284). This was a limitation which was beyond the authors, as the number of participants would depend on the eligibility criteria and voluntariness of individuals to be part of the study. This limitation was stated by the authors in the results and conclusion. Some other valid limitations which could affect the outcome of the study were stated in the last paragraph of "Discussion" section.

The limitations are presented in the last paragraph of the “Discussion section”

• The discussion was fraught with some vague statements and assertions not backed up by correct statistical analyses and references (Please see track changes in reviewed manuscript).
• Some of the figures (i.e., %) given in Tables 1-3 are wrong (Please see track changes in reviewed manuscript for corrections).
• Some revisions to the Tables have also been suggested.

All figures in table 1 have been verified, all are correct.

Figures on tables 2 and 3 have been verified and corrected. There were typos so statistical test values were not affected by the corrections. All other tables were verified.

6. PLOS authors have the option to publish the peer review history of their article ([what does this mean?](https://journals.plos.org/plosone/s/editorial-and-peer-review-process#loc-peer-review-history)). If published, this will include your full peer review and any attached files.


If you choose “no”, your identity will remain anonymous but your review may still be made public.


**Do you want your identity to be public for this peer review?** For information about this choice, including consent withdrawal, please see our [Privacy Policy](https://www.plos.org/privacy-policy).

Reviewer #1: No

Reviewer #2: No

[NOTE: If reviewer comments were submitted as an attachment file, they will be attached to this email and accessible via the submission site. Please log into your account, locate the manuscript record, and check for the action link "View Attachments". If this link does not appear, there are no attachment files.]

While revising your submission, please upload your figure files to the Preflight Analysis and Conversion Engine (PACE) digital diagnostic tool, <https://pacev2.apexcovantage.com/>. PACE helps ensure that figures meet PLOS requirements. To use PACE, you must first register as a user. Registration is free. Then, login and navigate to the UPLOAD tab, where you will find detailed instructions on how to use the tool. If you encounter any issues or have any questions when using PACE, please email PLOS at [figures@plos.org](mailto:figures@plos.org). Please note that Supporting Information files do not need this step.
