# Supplementary material for: Low levels of Plasmodium falciparum parasitaemia among patients receiving antiretroviral therapy at the treatment centre of the Regional Hospital Bamenda, Northwest Cameroon
Source: PLoS One. 2025 Jan 29;20(1):e0317120. doi: 10.1371/journal.pone.0317120 (PMC11778629; doi:10.1371/journal.pone.0317120)
Supplement: Attachment — Submitted filename: Response to reviewers.docx [file pone.0317120.s005.docx]

PONE-D-24-04976R1

Low levels of Plasmodium falciparum parasitemia among patients receiving antiretroviral therapy at the treatment center of the Regional Hospital Bamenda, Northwest Cameroon

PLOS ONE

Dear Dr. CALVIN EBAI,

Once again, thank you for submitting your manuscript to PLOS ONE. The manuscript has undergone a second Review and most of the initial comments have been addresed. However, there are some aspects that still need minor correcttions as highlighted below by one of the Reviewers. Kindly address these aspects as soon as possible.

We invite you to submit a revised version of the manuscript that addresses the points raised during the review process.

Please submit your revised manuscript by Dec 13 2024 11:59PM. If you will need more time than this to complete your revisions, please reply to this message or contact the journal office at [plosone@plos.org](mailto:plosone@plos.org). When you're ready to submit your revision, log on to https://www.editorialmanager.com/pone/ and select the 'Submissions Needing Revision' folder to locate your manuscript file.

Please include the following items when submitting your revised manuscript:

- A rebuttal letter that responds to each point raised by the academic editor and reviewer(s). You should upload this letter as a separate file labeled 'Response to Reviewers'
- A marked-up copy of your manuscript that highlights changes made to the original version. You should upload this as a separate file labeled 'Revised Manuscript with Track Changes'.
- An unmarked version of your revised paper without tracked changes. You should upload this as a separate file labeled 'Manuscript'.

If you would like to make changes to your financial disclosure, please include your updated statement in your cover letter. Guidelines for resubmitting your figure files are available below the reviewer comments at the end of this letter.

If applicable, we recommend that you deposit your laboratory protocols in protocols.io to enhance the reproducibility of your results. Protocols.io assigns your protocol its own identifier (DOI) so that it can be cited independently in the future. For instructions see: [https://journals.plos.org/plosone/s/submission-guidelines#loc-laboratory-protocols](https://track.editorialmanager.com/CL0/https:%2F%2Fjournals.plos.org%2Fplosone%2Fs%2Fsubmission-guidelines%23loc-laboratory-protocols/1/010f0192d9937c4a-1c91f723-0c59-4ad0-8a02-f55aaffdb457-000000/ZGzMOTFqmbHW6UDTmONQP-0imvSuohYOcJ-KWElTyKA=183). Additionally, PLOS ONE offers an option for publishing peer-reviewed Lab Protocol articles, which describe protocols hosted on protocols.io. Read more information on sharing protocols at [https://plos.org/protocols?utm_medium=editorial-email&utm_source=authorletters&utm_campaign=protocols](https://track.editorialmanager.com/CL0/https:%2F%2Fplos.org%2Fprotocols%3Futm_medium=editorial-email%26utm_source=authorletters%26utm_campaign=protocols/1/010f0192d9937c4a-1c91f723-0c59-4ad0-8a02-f55aaffdb457-000000/vS51iswKmx_hutx9TilR9S0O6nGh0xuuYV5eiRhzU8E=183).

We look forward to receiving your revised manuscript.

Kind regards,

Segun Isaac OYEDEJI, Ph.D
Academic Editor
PLOS ONE

Journal Requirements:

Please review your reference list to ensure that it is complete and correct. If you have cited papers that have been retracted, please include the rationale for doing so in the manuscript text, or remove these references and replace them with relevant current references. Any changes to the reference list should be mentioned in the rebuttal letter that accompanies your revised manuscript. If you need to cite a retracted article, indicate the article’s retracted status in the References list and also include a citation and full reference for the retraction notice.


[Note: HTML markup is below. Please do not edit.]

Reviewers' comments:

Reviewer's Responses to Questions: the authors want to sincerely thank the editors and the reviewers for the detailed critic of the paper to ensure the proper communication of this manuscript. Authors have taken out time to do the recommended corrections.

**Comments to the Author**

1. If the authors have adequately addressed your comments raised in a previous round of review and you feel that this manuscript is now acceptable for publication, you may indicate that here to bypass the “Comments to the Author” section, enter your conflict of interest statement in the “Confidential to Editor” section, and submit your "Accept" recommendation.

Reviewer #1: All comments have been addressed

Reviewer #2: (No Response)

2. Is the manuscript technically sound, and do the data support the conclusions?

The manuscript must describe a technically sound piece of scientific research with data that supports the conclusions. Experiments must have been conducted rigorously, with appropriate controls, replication, and sample sizes. The conclusions must be drawn appropriately based on the data presented.

Reviewer #1: Yes

Reviewer #2: Partly

3. Has the statistical analysis been performed appropriately and rigorously?

Reviewer #1: Yes

Reviewer #2: Yes

4. Have the authors made all data underlying the findings in their manuscript fully available?

The [PLOS Data policy](http://track.editorialmanager.com/CL0/http:%2F%2Fwww.plosone.org%2Fstatic%2Fpolicies.action%23sharing/1/010f0192d9937c4a-1c91f723-0c59-4ad0-8a02-f55aaffdb457-000000/aAkXjFaMVJgg9APzjPtAYQJnulIDThSSTgYtjzcqCck=183) requires authors to make all data underlying the findings described in their manuscript fully available without restriction, with rare exception (please refer to the Data Availability Statement in the manuscript PDF file). The data should be provided as part of the manuscript or its supporting information, or deposited to a public repository. For example, in addition to summary statistics, the data points behind means, medians and variance measures should be available. If there are restrictions on publicly sharing data—e.g. participant privacy or use of data from a third party—those must be specified.

Reviewer #1: Yes

Reviewer #2: No

5. Is the manuscript presented in an intelligible fashion and written in standard English?

PLOS ONE does not copyedit accepted manuscripts, so the language in submitted articles must be clear, correct, and unambiguous. Any typographical or grammatical errors should be corrected at revision, so please note any specific errors here.

Reviewer #1: Yes

Reviewer #2: No

6. Review Comments to the Author

Please use the space provided to explain your answers to the questions above. You may also include additional comments for the author, including concerns about dual publication, research ethics, or publication ethics. (Please upload your review as an attachment if it exceeds 20,000 characters)

Reviewer #1: (No Response)

Reviewer #2: Please find below, detailed comments on the clean copy of the revised manuscript.
General:
• The English language quality (including punctuations, etc.) just average and needs thorough editing and improvement.

Thorough editing has been done and corrections effected.

There is still a need for consistency in spellings throughout the manuscript; examples include dyspnea/dyspnoea; diarrhea/diarrhoea; parasitemia/parasitaemia at different points in the manuscript. These were pointed out in the earlier review.

Agreed. The spellings have been harmonised.

• There is a mix-up of in-text citations and corresponding references, some of which are listed below. Hence, there is a need for a painstaking audit and reconciliation of all in-text citations and corresponding references to ensure correctness.

Agreed. There has been a complete review of the references, both in-text and in the list of references.

Abstract:
Line 15: ART … is undefined. It would be necessary to have this acronym preceded by its full meaning while ART is placed in parentheses, i.e., “Antiretroviral therapy (ART)”. The acronym suffices, subsequently.

The full meaning of ART has been written and the acronym inserted in parenthesis.

Introduction:
• There is a need to underpin the statement in lines 67-70: “The parasite, especially P. falciparum, is associated with immunosuppression and massive destruction of red blood cells leading to anaemia, asthenia, respiratory difficulties and cerebral malaria, in case parasites are sequestrated in deep veins of the brain, among other manifestations.” with relevant citation(s)/reference(s) as suggested in the earlier review.

Agreed. Relevant references have been cited.

• Line 72: Replace “for which” with “in whom”: Agreed. Done.

• Lines 73-74: Recast as “However, an efficient management of the comorbidities is key to averting this severity.” Done.
• Lines 75-77: The statement preceding the in-text citation [15] does not align with the citation. The statement is underpinned more by [14] (please refer to lines 379-380 under References). The authors need to reconcile this. Agreed, corrected.
• Lines 81-83: The statement “HIV transmission is principally through unprotected sexual intercourse, but also through transfusion of infected blood, intravenous (IV) drug use and from mother to child, especially during childbirth, as well as accidental exposure to infected blood [17].”) does not align with the citation [17]. The publication [i.e., 17] was on CD4+ and nowhere in the article was reference made to HIV transmission. Agreed, the appropriate reference has been cited.

• Lines 94-95: The statement “In Cameroon, an estimated 480 000 people are infected with HIV, 9, 900 new cases of infection were recorded in 2023 with about 2.6% (12,480) deaths [21].” The citation may need to be reconciled with both [21] and [22] under References for correctness. Agreed, the correct references have been inserted.
• Line 197: [15] is a wrong citation for the preceding statement which aligns more with [14] under References (please refer to lines 379-380). Agreed, correction has been done.

• Line 104: [15] is a wrong citation for the preceding statement which is on HIV. [15] is a study on malaria! Agreed. Correction has been done.
• Lines 110-111: [19], [20], and [21] are wrong citations. The sentence in these lines align more with [23], [24] and [25], respectively. Please double-check for correctness. Agreed and revised.

Materials and Methods
Line 135: The in-text citations [26,27] do not align with the preceding statement but more with [27,28] (please refer to References). Please reconcile/double-check. Agreed. Reconciled.
Lines 147-148: [25] does not align with Cochran formula which can be found in [29] under References. Agreed, corrected.
Line 150: [21] does not align with any study in Bamenda by Eyong et al. This citation, i.e., [21] is a World Health Organization publication. The mistake has been corrected.
Lines 154-157: Recast the sentence to “After explaining the study objectives and procedures to eligible participants, a pretested questionnaire was administered to those who consented in order to obtain data on socio-demographic characteristics, clinical manifestations, history of ART, environmental factors and the methods of malaria prevention.” Agreed. Sentence has been recast.
Line 162: … to disinfect the tip of the finger before pricking. Which of the fingers? The authors need to state this. That has been added.
Lines 168 and 175: [29] does not align with the preceding statement. This citation, i.e., [29] is “Cochran WG. Sampling Techniques, John Wiley and Sons Inc., New York; 1963.” Agreed and corrected.
Line 171: delete “)”: Done
Results
Table 1: Under religion, None, the 5.6 should be 5.5. Please note that 10/181 = 5.52486. This was pointed out in the earlier review. Corrected
Table 2: Under χ2 (p-value) for Type of house, amend χ2 =0.27 to 0.27 (0.61). Corrected.

Discussion
Lines 265-266: [19] and [21] do not align with Kimbi et al. and Achere et al., respectively, as stated. They are both WHO publications, while Kimbi et al. and Achere et al. are placed as [23] and [25], respectively (please refer to References). Corrected.
Lines 268-269: Please recast to “combat the disease [31]. Moreover, in the past, ART was not free while there has been a lot of education through the mass media, especially in hospital settings. This might have benefited the masses”. Agreed. Sentence has been recast.
Line 271: Please recast to “2020 due to a breakdown in health systems as a result of the Covid 19 pandemic [???].” Agreed. Corrected.
Line 271: [17] does not align with the preceding statement. Agree. The correct reference has been inserted.
Line 273: [20] is not Njunda et al. in 2012, in References. It is actually [24]. Corrected
Line 275: [30] is not Akinbo et al. in 2016, in References. It is actually [31]. Corrected
Line 280: [33] and [34] are not Sahle et al. and Simon-Oke et al. respectively, in References. They are actually [34] and [35], respectively. Corrected.
Ditto, all in-text citations and corresponding references onward. The authors need to do a comprehensive review of the in-text citations so as to correspond with their listing in References. Agreed. This has been done.
Lines 305-307: Please recast to: “Studies have shown varied reports on this; Jegede et al. [39] showed no significant statistical difference while Akinbo et al. [31] reported significant statistical difference.” Corrected
Line 316: Please amend as follows: “and non-salary earnings which are generally associated with some physical activity that may contribute” Agreed and amended.

References
The correct citations are as follows:
1. Global Health Estimates 2020: Deaths by Cause, Age, Sex, by Country and by Region, 2000-2019. Geneva, World Health Organization; 2020.
37. Gumel SD, Ibrahim A, Olayinka AT, Ibrahim MS, Balogun MS, Dahiru A, et al. HIV-malaria 450 co-infection and its determinants among patients attending antiretroviral treatment 451 clinic in Zaria, Kaduna State, Nigeria. Journal of Interventional Epidemiology and Public Health, 2021; 4: 2. DOI: https://doi.org/10.37432/jieph.2021.4.1.31.
A click at the above DOI shows the publication titled as above. The DOI link does not correspond to the citation provided by the authors. The authors may wish to access the link for correctness.
38. Tchinda GG, Atashili J, Achidi EA, Kamga HL, Njunda AL, Ndumbe PM. Impact of Malaria on Hematological Parameters in People Living with HIV/AIDS Attending the Laquintinie Hospital in Douala, Cameroon. PLoS ONE, 2012; 7(7): e40553. https://doi.org/10.1371/journal.pone.0040553.
A visit to the link provided in the citation by the authors (i.e., Available from: https://www.ncbi.nlm.nih.gov/pmc/articles/PMC3393653/https://doi.org/10.1371/journal.pone.0040553)., shows that the proper citation is as stated above.

The citations (1, 37 38) and the others have all been corrected.

• Line 392: Amend manifestation to manifestations. Done
• Line 394: Amend https://doi.org/10.172226/938. to <https://doi.org/10.17226/938>. Done
• Line 421: the correct citation is: Sci Rep. 2021, 11: 16655. DOI:10.1038/s41598-021-95591-6. Done
• Lines 446-447: the correct citation is …. 44: 156. DOI:10.1186/s42269-020-00401-0. Done
• Lines 464 and 468: Amend PLOS to PLoS. Corrected
