# Supplementary material for: Low levels of Plasmodium falciparum parasitaemia among patients receiving antiretroviral therapy at the treatment centre of the Regional Hospital Bamenda, Northwest Cameroon
Source: PLoS One. 2025 Jan 29;20(1):e0317120. doi: 10.1371/journal.pone.0317120 (PMC11778629; doi:10.1371/journal.pone.0317120)
Supplement: Attachment — Submitted filename: REVIEWER COMMENTS.docx [file pone.0317120.s001.docx]

Dear Editor,

I refer you to the attached reviewed manuscript (please see track changes) for my detailed comments on all sections of the manuscript. Please find below, a few of the specific comments/concerns and suggestions:

- The conceptualization of the study was adequate but the execution was inadequate. The manuscript needs to be thoroughly revised to make for a better article. **Please see attached "Reviewed" manuscript.**
- The authors’ recourse to spelling of the word ‘’**Parasitemia’’** interchangeably with ‘’**Parasitaemia**’’ in the manuscript is not ideal. For consistency and depending on Journal style, the authors should adopt only one spelling; American (**parasitemia**) or British (**parasitaemia**).
- **Line 41**: Reference [1] is exclusive to the assertion on HIV as a leading cause of mortality. The authors need to back up a similar assertion on malaria with a reference.
- **Line 61**: The authors should double-check the highlighted figures/estimates (6.459.013 and 12.587) for correctness. Could be typos.
- **Lines 74/75**: The highlighted sentence is vague and needs to be recast for better comprehension.
- **Line 90**: The authors should please reconcile these estimates 89% (29.8 million).
- The objectives of the study were clearly stated and the study design described. However, the clinical manifestations and factors associated with malaria parasitaemia among HIV patients on ART, as described in the study, were not appropriate/adequate to address the stated objectives. Moreover,
- **Line 168/169**: Authors need to be more specific. They did not state whether or not the two drops of blood for preparation of blood smears were inclusive/exclusive of the very first drop of blood after pricking the finger with a sterile lancet?
- **Line 171**: ‘’Both thick and thin films were stained with 10% Giemsa stain …..’’
- Although this yields faster results, the films may be of less predictable quality than if stained with 2.5% or 3% Giemsa stain. One would have expected that for such a study, either of the latter concentrations would have been used.
- Actual sample size (181) was far less than the estimated sample size (284). This was a limitation which was beyond the authors, as the number of participants would depend on the eligibility criteria and voluntariness of individuals to be part of the study. This limitation was stated by the authors in the results and conclusion. Some other valid limitations which could affect the outcome of the study were stated in the last paragraph of "Discussion" section.
- The discussion was fraught with some vague statements and assertions not backed up by correct statistical analyses and references (Please see track changes in reviewed manuscript).
- Some of the figures (i.e., **%**) given in Tables 1-3 are wrong (Please see track changes in reviewed manuscript for corrections).
- Some revisions to the Tables have also been suggested.
